# Real-World Data on Cabozantinib in Previously Treated Patients with Metastatic Renal Cell Carcinoma: Focus on Sequences and Prognostic Factors

**DOI:** 10.3390/cancers12010084

**Published:** 2019-12-30

**Authors:** Matteo Santoni, Daniel Y. Heng, Sergio Bracarda, Giuseppe Procopio, Michele Milella, Camillo Porta, Marc R. Matrana, Giacomo Cartenì, Simon J. Crabb, Ugo De Giorgi, Umberto Basso, Cristina Masini, Fabio Calabrò, Maria Giuseppa Vitale, Daniele Santini, Francesco Massari, Luca Galli, Giuseppe Fornarini, Riccardo Ricotta, Sebastiano Buti, Paolo Zucali, Orazio Caffo, Franco Morelli, Francesco Carrozza, Angelo Martignetti, Alain Gelibter, Roberto Iacovelli, Alessandra Mosca, Francesco Atzori, Nuno Vau, Lorena Incorvaia, Cinzia Ortega, Marina Scarpelli, Antonio Lopez-Beltran, Liang Cheng, Vittorio Paolucci, Jeffrey Graham, Erin Pierce, Sarah Scagliarini, Pierangela Sepe, Elena Verzoni, Sara Merler, Mimma Rizzo, Giulia Sorgentoni, Alessandro Conti, Francesco Piva, Alessia Cimadamore, Rodolfo Montironi, Nicola Battelli

**Affiliations:** 1 Oncology Unit, Macerata Hospital, via Santa Lucia 2, 62100 Macerata, Italy; mattymo@alice.it (M.S.); vittorio.paolucci@sanita.marche.it (V.P.); giulia.sorgentoni@libero.it (G.S.); 2 Division of Medical Oncology, Department of Oncology, Tom Baker Cancer Centre, University of Calgary, Calgary, AB T2N 4N2, Canada; Daniel.Heng@albertahealthservices.ca (D.Y.H.); jeffgraham1@gmail.com (J.G.); 3 Medical Oncology, Department of Oncology, AziendaOspedaliera S. Maria, 05100 Terni, Italy; sergio.bracarda@gmail.com; 4 Department of Medical Oncology, Istituto Nazionale deiTumori IRCCS, 20133 Milan, Italy; giuseppe.procopio@istitutotumori.mi.it (G.P.); Pierangela.Sepe@istitutotumori.mi.it (P.S.); Elena.Verzoni@istitutotumori.mi.it (E.V.); 5 U.O.C. Oncology, AziendaOspedalieraUniversitariaIntegrata, University and Hospital Trust of Verona, 37126 Verona, Italy; michele.milella@univr.it (M.M.); saramerler@yahoo.it (S.M.); 6 Department of Internal Medicine and Therapeutics, University of Pavia and Division of Translational Oncology, IRCCS Istituti Clinici Scientifici Maugeri, 27100 Pavia, Italy; camillo.porta@gmail.com (C.P.); rizzo.mimma@gmail.com (M.R.); 7 Department of Internal Medicine, Hematology/Oncology, Ochsner Medical Center, New Orleans, LA 70121, USA; mamatrana@ochsner.org (M.R.M.); epierce@ochsner.org (E.P.); 8 Department of Medical Oncology, AO “A. Cardarelli”, 80131 Naples, Italy; cartenigiacomo@gmail.com (G.C.); sarahscagliarini@gmail.com (S.S.); 9 Cancer Sciences Unit, University of Southampton, Southampton SO171BJ, UK; S.J.Crabb@southampton.ac.uk; 10 Department of Medical Oncology, IstitutoScientifico Romagnolo per lo Studio e la CuradeiTumori (IRST) IRCCS, 47014 Meldola, Italy; ugo_degiorgi@yahoo.com; 11 Department of Medical Oncology, IstitutoOncologico Veneto (IOV) IRCCS, 35128 Padova, Italy; umberto.basso@iov.veneto.it; 12 Medical Oncology Unit, Arcispedale Santa Maria Nuova, IRCCS Reggio Emilia, 42123 Reggio Emilia, Italy; cristina.masini2@asmn.re.it; 13 San Camillo-Forlanini Hospital, 00128 Rome, Italy; fabiocalabro1@gmail.com; 14 Department of Oncology and Haematology and Respiratory Disease, University Hospital, 41125 Modena, Italy; vitalemariag@gmail.com; 15 Department of Medical Oncology, Campus Bio-Medico University of Rome, 00128 Rome, Italy; 16 Division of Oncology, S. Orsola-Malpighi Hospital, 40138 Bologna, Italy; fmassari79@gmail.com; 17 Medical Oncology Unit, Department of Translational Research and New Technologies in Medicine, University of Pisa, 56126 Pisa, Italy; lugal71@yahoo.it; 18 Department of Medical Oncology, Ospedale “S. Martino”, 16132 Genova, Italy; giuseppe.fornarini@hsanmartino.it; 19 Niguarda Cancer Center, Grande OspedaleMetropolitano Niguarda, 20162 Milan, Italy; riccardo.ricotta@ospedaleniguarda.it; 20 Medical Oncology Unit, University Hospital of Parma, 43126 Parma, Italy; sebabuti@libero.it; 21 Humanitas Clinical and Research Center, Humanitas Cancer Center, Rozzano, 20089 Milano, Italy; paolo.zucali@humanitas.it; 22 Medical Oncology Department, Santa Chiara Hospital, Largo Medaglied’Oro, 38122 Trento, Italy; orazio.caffo@apss.tn.it; 23 Medical Oncology Department, Casa SollievodellaSofferenza, VialeCappuccini 1, 71013 San Giovanni Rotondo, Italy; f.morelli@operapadrepio.it; 24 Oncology Unit, City Hospital, 48018 Faenza, Italy; fcarroyya@yahoo.it; 25 Dipartimentooncologicouslsud-esttoscana-area senese, LocalitàCampostaggias.n.c., 53036 Poggibonsi, Italy; angelo.martignetti@uslsudest.toscana.it; 26 Medical Oncology (B), Policlinico Umberto I, “Sapienza” University of Rome, 00128 Rome, Italy; agelibter@yahoo.it; 27 Medical Oncology, Fondazione Policlinico Universitario A. Gemelli IRCCS, 00168 Roma, Italy; roberto.iacovelli@policlinicogemelli.it; 28 Medical Oncology Unit, Maggiore dellaCarità University Hospital, University of Eastern Piedmont, 28100 Novara, Italy; alessandramosca25@yahoo.it; 29 Medical Oncology Unit, AziendaOspedalieroUniversitaria of Cagliari, 09124 Cagliari, Italy; francescoatzori74@yahoo.it; 30 Urologic Oncology, Champalimaud Clinical Center, 1400-038 Lisbon, Portugal; nuno.vau@fundacaochampalimaud.pt; 31 Department of Surgical, Oncological and Oral Sciences, Section of Medical Oncology, University of Palermo, 90127 Palermo, Italy; lorena.incorvaia@unipa.it; 32 Department of Medical Oncology, Ospedale S. Lazzaro ASL CN2 Alba-Bra, 12051 Cuneo, Italy; cinzia.ortega@gmail.com; 33 United Hospitals, School of Medicine, Section of Pathological Anatomy, Polytechnic University of the Marche Region, Via Conca 71, I-60126 Ancona, Italy; m.scarpelli@univpm.it (M.S.); alessiacimadamore@gmail.com (A.C.); 34 Department of Pathology and Surgery, Faculty of Medicine, 14004 Cordoba, Spain; em1lobea@gmail.com; 35 Department of Pathology and Laboratory Medicine, Indiana University School of Medicine, Indianapolis, IN 46202, USA; liang_cheng@yahoo.com; 36 Department of Urology, Bressanone/Brixen hospital, via Dante 51, 39042 Bressanone BZ, Italy; alessandro.conti83@gmail.com; 37 Department of Specialistic Clinical and Odontostomatological Sciences, Polytechnic University of Marche, 60126 Ancona, Italy; f.piva@univpm.it

**Keywords:** cabozantinib, nivolumab, prognosis, real-world data, renal cell carcinoma, targeted therapy

## Abstract

Cabozantinib is approved for the treatment of renal cell carcinoma (RCC). However, prognostic factors are still lacking in this context. The aim of this study was to evaluate prognostic factors in RCC patients treated with second- or third-line cabozantinib. A multicenter retrospective real-world study was conducted, involving 32 worldwide centers. A total of 237 patients with histologically confirmed clear-cell and non-clear-cell RCC who received cabozantinib as second- or third-line therapy for metastatic disease were included. We analyzed overall survival (OS), progression-free survival (PFS) and time-to-strategy failure (TTSF) using Kaplan–Meier curves. Cox proportional models were used at univariate and multivariate analyses.The median PFS and OS of cabozantinib were 7.76 months (95% CI 6.51–10.88) and 11.57 months (95% CI 10.90–not reached (NR)) as second-line and 11.38 months (95% CI 5.79–NR) and NR (95% CI 11.51–NR) as third-line therapy. The median TTSF and OS were 11.57 and 15.52 months with the sequence of cabozantinib–nivolumab and 25.64 months and NR with nivolumab–cabozantinib, respectively. The difference between these two sequences was statistically significant only in good-risk patients. In the second-line setting, hemoglobin (Hb) levels (HR= 2.39; 95% CI 1.24–4.60, *p* = 0.009) and IMDC (International Metastatic Renal Cell Carcinoma Database Consortium) group (HR = 1.72, 95% CI 1.04–2.87, *p* = 0.037) were associated with PFS while ECOG-PS (HR = 2.33; 95%CI, 1.16–4.69, *p* = 0.018) and Hb levels (HR = 3.12; 95%CI 1.18–8.26, *p* = 0.023) correlated with OS at multivariate analysis, while in the third-line setting, only Hb levels (HR = 2.72; 95%CI 1.04–7.09, *p* = 0.042) were associated with OS. Results are limited by the retrospective nature of the study.This real-world study provides evidence on the presence of prognostic factors in RCC patients receiving cabozantinib.

## 1. Introduction

Renal cell carcinoma (RCC), the most common kidney cancer in adults, represents 5% of all cancers in men and 3% in women, with an estimated 65,340 new cases and 14,970 deaths in 2018 in the United States alone [1]. Agents able to target altered pathways promoting neoangiogenesis (e.g., sunitinib, pazopanib, sorafenib, axitinib and tivozanib [2,3,4,5,6,7,8,9]) have demonstrated activity in metastatic RCC (mRCC), as well as immunecheckpoint inhibitors used alone as nivolumab [10] or combined with other immunotherapy (nivolumab plus ipilimumab [11]) or targeted therapies (axitinib plus pembrolizumab or avelumab) [12,13].

Cabozantinib is an orally administered tyrosine kinase inhibitor acting mainly on VEGFR2, MET (mesenchymal epithelial transition receptor) and AXL (anexelekto pathway) [14]. In the randomized phase III METEOR trial comparing cabozantinib to everolimus in pretreated patients, cabozantinib improved overall survival (OS), progression-free survival (PFS) and objective response rate (ORR) [15]. Serious adverse events with cabozantinib occurred in 39% of patients, with the most common toxicities being hypertension, diarrhea and fatigue [16]. 

Most recently, a randomized phase II clinical trial (CABOSUN) randomized 157 patients with mRCC and intermediate or poorrisk of disease according to IMDC (International Metastatic Renal Cell Carcinoma Database Consortium) criteria (based on the presence of anemia, neutrophilia, thrombocytosis, Karnofskyperformance status <80, hypercalcemia and <1 year from diagnosis to metastatic disease) to receive cabozantinib or sunitinib as first-line therapy [17]. Compared to sunitinib, cabozantinib improved PFS and ORR in this subgroup of patients. Despite the fact that these findings were also confirmed on a subsequent analysis based on independent review [18], the results of this study are still controversial [19].

To date, cabozantinib is indicated for the treatment of patients with advanced RCC in treatment-naïve adults with intermediate poor-risk features (Food and Drug Administration, FDA), and by EMA (European Medical Agency) for adults progressed to prior vascular endothelial growth factor/receptor inhibitors. Here, we report results of a real-world analysis on cabozantinib in previously treated patients with mRCC andaimed to evaluate the presence of prognostic factors and the different therapeutic sequences in this setting.

## 2. Results

### 2.1. Overall Population

A total of 237 patients were included in this analysis; 174 (73.42%) were males and 63 (26.58%) females. The median age was 62.56y (range 24.55–85.76). The majority of patients had clear-cell RCC (182 patients, 76.79%), while in 55 patients (23.21%) non-clear-cell RCC (17 papillary type I, 14 papillary type II, 14 clear-cell RCC with sarcomatoid differentiation, 1 with rhabdoid differentiation, 1 chromophobe, 1 with XP11.3 translocation and 7 unclassified RCC tumors) was diagnosed. There were 120 patients (50.63%) who were metastatic at time of diagnosis. At first diagnosis, the Fuhrman or WHO/ISUP grade was G3 in 86 (36.29%) and G4 in 32 (13.50%). The number of metastatic sites was ≥2 in 160 cases (67.51%). The most frequent sites of metastasis were lung (154 patients, 64.98%), lymph nodes (133 patients, 56.12%) and bone (80 patients, 34.04%). According to IMDC criteria, 57 patients (24.05%) were at favorable-risk, 146 (61.60%) at intermediate-risk and 34 (14.35%) had poor-risk features. Patients’ characteristics are reported in Table 1. The distribution of IMDC criteria across the study population is showed in Table 2.

The median follow-up time from diagnosis was 182.79 months (95% CI 131.00 to not reached;NR) and median OS from the start of first-line therapy was 103.23 months (95% CI 63.40–NR). During the follow-up, 73 patients (30.80%) died. A further 112 patients (47.26%) were treated with cabozantinib as second-line therapy, while 125 (52.74%) received cabozantinib in the third-line setting. In 41 patients, second-line cabozantinib was ongoing at the time of data collection. Among the 71 patients who progressed on second-line cabozantinib, 53 (74.65%) received a third-line therapy, which was nivolumab in 29 patients (54.72%). Drug distribution and sequence is reported in Table 1.

### 2.2. Progression-Free Survival of Cabozantinib as Second-Line Therapy

The median PFS of cabozantinib as second-line therapy was 7.76 months (95% CI 6.51–10.88, Table 3). 

**Figure 1:**
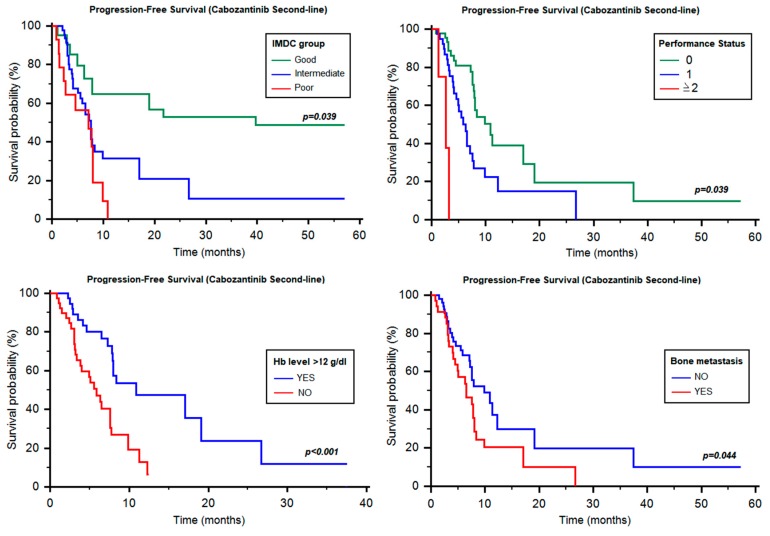
Progression-free survival of second-line cabozantinib according to different prognostic factors. Hb = hemoglobin; IMDC = International Metastatic Renal Cell Carcinoma Database Consortium.

This figure significantly changed when patients were analyzed according to their IMDC status, yielding 11.28 months (95% CI 7.89–NR, Table 3, Figure 1) in good-risk, 7.59 months (95% CI 5.52–NR, Table 3, Figure 1) in intermediate-risk and 7.13 months (95% CI 2.66–NR, Table 3, Figure 1) in poor-risk patients (*p* = 0.039). Similarly, PFS was different according to ECOG-performance status (PS; 0 vs. 1 vs. ≥2; 10.88 months vs. 5.88 months vs. 2.66 months, *p* < 0.001, Figure 1) and hemoglobin (Hb) ≥12 g/dL vs. <12 g/dL (10.88 vs. 5.88 months, HR = 0.39, 95% CI 0.18–0.62, *p* < 0.001, Figure 1). Otherwise, no significant difference was found based on time from diagnosis to systemic therapy (≥1y vs. <1y, 11.28 vs. 7.13 months, HR = 0.62, 95% CI 0. 73–1.14, *p* = 0.130), neutrophilia (7.76 vs. 4.01 months, HR = 0.48, 95% CI 0.13–1.01, *p* = 0.051), thrombocytosis (7.89 vs. 6.51 months, HR = 0.50, 95% CI 0.15–1.02, *p* = 0.055) and hypercalcemia (7.82 vs. 3.06 months, HR = 0.50, 95% CI 0.12–1.22, *p* = 0.106).

Interestingly, no significant differences were also found between clear-cell and non-clear-cell histology (7.89 vs. 5.06 months, HR = 0.73, 95% CI 0.35–1.40, *p* = 0.310), age < 70y and ≥70y (7.89 vs. 7.13 months, HR = 0.74, 95% CI 0.37–1.41, *p* = 0.334), gender (*p* = 0.678), Fuhrman or WHO/ISUP grade (*p* = 0.756) or number of metastatic sites (1 site vs. ≥2 sites, 7.59 vs. 7.82 months, HR = 0.99, 95% CI 0.56–1.76, *p* = 0.987).

By stratifying patients based on the site of metastasis, a significant difference was found between patients with or without bone metastases (6.51 vs. 9.86 months, HR = 0.58, 95% CI 0.31–0.98, *p* = 0.044, Figure 1), whilst no differences were found between patients with lung (6.05 vs. 6.31 months, HR = 0.88, 95% CI 0.64–1.21, *p* = 0.446), liver (7.59 vs. 12.3 months, HR = 1.48, 95% CI 0.73–2.81, *p* = 0.297), lymph node (7.59 vs. 7.89 months, HR = 1.23, 95% CI 0.71–2.16, *p* = 0.447), or brain metastases (7.76 vs. 7.59 months, HR = 1.24, 95% CI 0.52–2.89, *p* = 0.638). 

Furthermore, we analyzed the eventual prognostic role of the received first-line therapy, with any significant difference between sunitinib and pazopanib (7.89 vs. 7.82 months, HR = 1.25, 95% CI 0.70–2.38, *p* = 0.418).

Univariate analysis showed that ECOG-PS (HR = 2.47; 95% CI, 1.40–4.36, *p* = 0.002), Hb levels (HR = 2.90; 95% CI, 1.55–5.42, *p* < 0.001), IMDC group (HR = 1.77; 95% CI, 1.12–2.80, *p* = 0.015) and bone metastases (HR = 1.75; 95% CI, 1.10–3.02, *p* = 0.047) were significantly associated with the PFS of cabozantinib, given as second-line therapy. At multivariate analysis, only Hb levels (HR = 2.39; 95% CI, 1.24–4.60, *p* = 0.009) and IMDC group (HR = 1.72, 95% CI, 1.04–2.87, *p* = 0.037) maintained their prognostic significance in this setting.

### 2.3. Overall Survival of Cabozantinib as Second-Line Therapy

**Figure 2:**
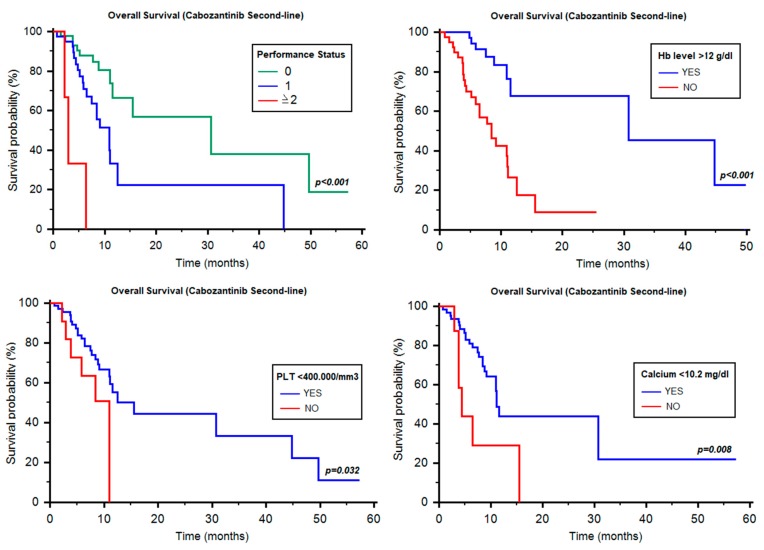
Overall survival of second-line cabozantinib according to different prognostic factors. Hb = hemoglobin.

The median OS of cabozantinib as second-line therapy was 11.57 months (95% CI 10.90–NR, Table 3). Differently from PFS, IMDC classification was not associated with OS in the three prognostic groups (12.53 vs. 10.95 vs. 11.05 months, *p* = 0.349, Table 3). Conversely, the median OS was significantly different according to ECOG-PS (0 vs. 1 vs. ≥2; 30.71 months vs. 10.95 months vs. 2.96 months, *p* < 0.001, Figure 2), Hb ≥12 g/dL vs. <12 g/dL (30.71 vs. 8.42 months, HR = 0.24, 95% CI 0.10–0.44, *p* < 0.001, Figure 2), thrombocytosis (15.52 vs. 10.95 months, HR = 0.42, 95% CI 0.09–0.90, *p* = 0.032, Figure 2) and hypercalcemia (11.08 vs. 4.37 months, HR = 0.32, 95% CI 0.04–0.60, *p* = 0.008, Figure 2). Of note, no significant differences were found for neutrophilia (12.53 vs. 11.57 months, HR = 0.57, 95% CI 0.17–1.48, *p* = 0.211), time from diagnosis to systemic therapy (≥1y vs. <1y, 11.57 vs. 11.05 months, HR = 1.02, 95% CI 0.51–2.07, *p* = 0.949), clear-cell and non-clear-cell histology (11.57 months vs. notreached (NR), HR = 0.83, 95% CI 0.33–1.98, *p* = 0.648), age < 70y and ≥70y (11.57 vs. 11.08 months, HR = 0.93, 95% CI 0.42–2.04, *p* = 0.856), Fuhrman grade (*p* = 0.899), choice of first-line therapy (sunitinib vs.pazopanib: 15.52 vs. 11.08 months, HR = 1.44, 95% CI 0.71–3.26, *p* = 0.281), site of metastasis and number of metastatic sites (1 site vs. ≥2 sites, 15.52 vs. 11.05 months, HR = 0.82, 95% CI 0.41–1. 46, *p* = 0.573).

At univariate analysis, ECOG-PS (HR = 3.51; 95% CI, 1.86–6.63, *p* < 0.001), Hb levels (HR = 5.07; 95% CI, 2.18–11.76, *p* < 0.001), thrombocytosis (HR = 2.52; 95% CI, 1.05–6.01, *p* = 0.039) and hypercalcemia (11.08 vs. 4.37 months, HR = 3.24, 95% CI 0.31–8.03, *p* = 0.015) were significant predictors of OS, while at multivariate analysis, only ECOG-PS (HR = 2.33; 95% CI, 1.16–4.69, *p* = 0.018) and Hb levels (HR = 3.12; 95% CI, 1.18–8.26, *p* = 0.023) correlated with OS.

### 2.4. Progression-Free Survival of Cabozantinib as Third-Line Therapy

**Figure 3:**
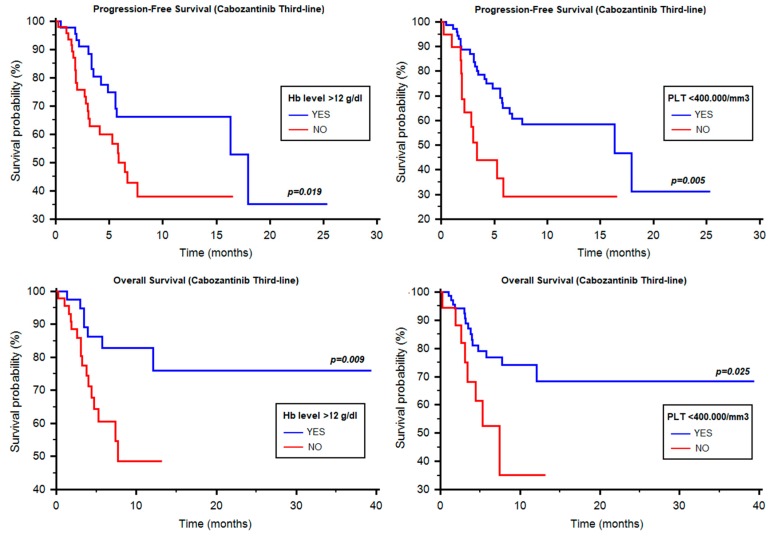
Progression-free survival and overall survival of third-line cabozantinib according to different prognostic factors. Hb = hemoglobin; PLT = platelets.

The median PFS of cabozantinib in the third-line setting was 11.38 months (95% CI 5.79–NR, Table 3). The median PFS was not statistically different among the three IMDC groups (11.38 vs. 7.63 vs. 5.75 months, *p* = 0.772, Table 3) or according to ECOG-PS (11.38 vs. 5.26 months, HR = 0.54, 95% CI 0.16–1.24, *p* = 0.120), time from diagnosis to systemic therapy (≥1y vs. <1y, NR vs. NR, HR = 0.57, 95% CI 0.27–1.35, *p* = 0.217), neutrophilia (7.63 months vs. NR, HR = 1.20, 95% CI 0.55–2.62, *p* = 0.657) and hypercalcemia (16.34 vs. 6.71 months, HR = 1.23, 95% CI 0.50–3.00, *p* = 0.652). Otherwise, PFS was statistically different according to Hb levels (17.95 vs. 6.44 months, HR = 0.47, 95% CI 0.24–0.88, *p* = 0.019, Figure 3) and thrombocytosis (16.34 vs. 3.35 months, HR = 0.39, 95% CI 0.12–0.68, *p* = 0.005, Figure 3). 

Of note, no significant differences were found by stratifying patients by clear-cell vs. non-clear-cell histology (6.71 vs. 11.38 months, HR = 1.26, 95% CI 0.61–2.59, *p* = 0.539), age < 70y and ≥70y (6.44 vs. 11.38 months, HR = 1.57, 95% CI 0.82–2.80, *p* = 0.183), Fuhrman grade (*p* = 0.474) or number or site of metastases.

Hb levels (HR = 2.19; 95% CI, 1.12–4.26, *p* = 0.022) and thrombocytosis (HR = 2.60; 95% CI, 1.30–5.19, *p* = 0.007), were significantly correlated with PFS at univariate but not at multivariate analysis.

### 2.5. Overall Survival of Cabozantinib as Third-Line Therapy

In the 125 patients treated with cabozantinib in third-line setting, the median OS was NR (95% CI 11.51–NR, Table 3). Hb ≥12 g/dL vs. <12 g/dL (NR vs. 7.73 months, HR = 0.33, 95% CI 0.14–0.76, *p* = 0.009, Figure 3), thrombocytosis (NR vs. 7.40 months, HR = 0.39, 95% CI 0.10–0.86, *p* = 0.025, Figure 3). Interestingly, no significant differences were found according to IMDC group (*p* = 0.739, Table 3), ECOG-PS (NR vs. 7.73 months, HR = 0.45, 95% CI 0.10–1.11, *p* = 0.073), neutrophilia (NR vs. NR months, HR = 0.73, 95% CI 0.26–1.97, *p* = 0.509), time from diagnosis to systemic therapy (NR vs. NR months, HR = 0.57, 95% CI 0.27–1.35, *p* = 0.217), hypercalcemia (NR vs. 7.40 months, HR = 0.73, 95% CI 0.21–2.34, *p* = 0.560), clear-cell vs. non-clear-cell histology (NR vs. NR, HR = 1.06, 95% CI 0.41–2.74, *p* = 0.906), age < 70y and ≥70y (12.10 months vs. NR, HR = 2.61, 95% CI 0.96–4.77, *p* = 0.063), Fuhrman or WHO/ISUP grade (*p* = 0.574) and neither specific sites nor number of metastatic sites (1 site vs. ≥2 sites, NR vs. NR, HR = 0.72, 95% CI 0.34–1.57, *p* = 0.422).

Fromunivariate analysis, Hb levels (HR = 3.11; 95% CI, 1.27–7.72, *p* = 0.014) and thrombocytosis (HR = 2.59; 95% CI, 1.10–6.13, *p* = 0.030) were found to be associated with OS, while for multivariate analysis, only Hb levels (HR = 2.72; 95% CI, 1.04–7.09, *p* = 0.042) were correlated with OS.

### 2.6. Time to Strategy Failure and Sequencing: Cabozantinib vs. Nivolumab

**Figure 4:**
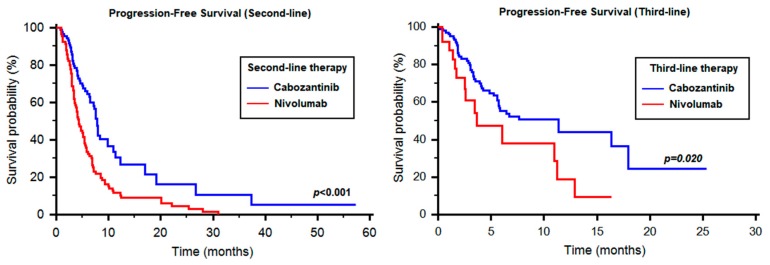
Comparison between cabozantinib and nivolumab in the second- and third-line setting.

In the 89 patients treated with second-line nivolumab, we observed a median PFS of 4.31 months (95% CI 3.65–5.46), which was significantly different from cabozantinib in the same setting (HR 0.50, 95% CI 0.35–0.69, *p* < 0.001, Figure 4). Similarly, the 29 patients treated with third-line nivolumab had a median PFS of 3.68 months vs. 11.38 months registered by cabozantinib (HR 0.50, 95% CI 0.19–0.87, *p* = 0.020, Figure 4). The median TTSF from the start of second-line therapy was 18.61 months (95% CI 16.30–21.80).

**Figure 5:**
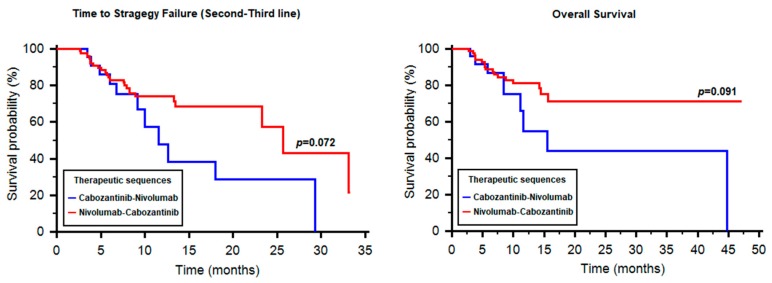
Time to strategy failure and overall survival in patients treated with the sequences cabozantinib–nivolumab and nivolumab–cabozantinib.

By stratifying patients according to the therapeutic sequence received, we reported 29 patients treated with cabozantinib followed by nivolumab and 89 patients who received nivolumab followed by cabozantinib. The median TTSF was 11.57 months with cabozantinib–nivolumab (95% CI 9.17–NR, Figure 5) and 25.64 months with nivolumab–cabozantinib (95% CI 23.24–NR, Figure 5). The difference was statistically significant only in the good-risk group (11.57 vs. 25.64 months, HR 8.99, 95% CI 3.91–671.63, *p* = 0.003).

The median OS from the start of second-line therapy in the cabozantinib–nivolumab and nivolumab–cabozantinib groups were 15.52 months (95% CI 11.10–NR) and NR (95% CI NR–NR), respectively (HR 1.95, 95% CI 0.87–5.83, *p* = 0.091, Figure 5). Similarly to TTSF, the median OS was significantly longer with nivolumab–cabozantinib only in the good-risk subgroup (13.55 months vs. NR, *p* = 0.004).

## 3. Discussion

Cabozantinib represents an effective strategy in untreated and pretreated RCC patients. The lack of validated molecular or clinical predictive and prognostic factors aimed to optimize the efficacy and safety of this agent represents a major challenge for uro-oncologists. In our analysis, only Hb levels were significantly correlated with OS in the second- and third-line settings. The negative prognostic significance of anemia suggests that a prompt management of this condition could have a potential impact on the outcome of RCC patients. Moreover, these data support the necessity of investigating the prognostic significance of anemia in patients treated with nivolumab to understand if this condition may represent a key factor in the decision-making process between these two agents.In particular, reversible causes of anemia need to be addressed, but the data do not indicate attempting erythropoietin(EPO)-induced correction, particularly given the association of EPO with adverse outcomes in cancersin large studies [20].

Interestingly, no difference in terms of efficacy has been found between clear-cell and non-clear-cell histologies. This may be partially explained by the prevalence in the non-clear-cell group of papillary tumors (31/55), in which cabozantinib has demonstrated to be effective [21]. 

There is a clear statistical difference between the two sequences in favorable-risk RCCpatients (*p* = 0.003); and for all patients analyzed, there is a trend towards statistical significance with a clear separation of curves for TTSF and OS beyond 12 months. Even though cabozantinibhas better PFSin the second-line setting than nivolumab (Figure 4), there appears to be utility in trying the sequencenivolumab–cabozantinib, rather than cabozantinib–nivolumab, particularly in favorable-risk patients, which needs to be further investigated with a larger sample size. These data also call for studies investigating the biological rationale for differences in outcomes between the sequences. However, the small number of patients in each prognostic group and the retrospective nature of our study do not allow to definitively clarify this issue. 

## 4. Patients and Methods

### 4.1. Study Population

The study population included adults (>18 years) with clear-cell or non-clear-cell mRCC, treated with cabozantinib as second- or third-line therapy. Patients were treated in 32 worldwide institutions between November 2004 and January 2019. Data were retrospectively collected from patients’ electronic medical records and paper charts. Patients were excluded from this study if they had missing data regarding thesite of metastasis and tumor response to therapy. The research was carried out in accordance with the approval by the ethical committee of the participating institutions. The study has been accepted by the “Comitato Etico Regionale delle Marche”, the accepting number is 2019-403.

### 4.2. Treatment Regimens and Statistical Analysis

Cabozantinib was administered orally, usually at a starting dose of 60 mg once daily. Treatment was administered until clinical or radiological disease progression, serious adverse events or death. Follow-up commonly consisted of periodic physical examination, laboratory analysis and imaging assessment by computed tomography (CT) or magnetic resonance imaging (MRI) not earlier than 4 weeks, and not later than 6 weeks, according to local regulations. Disease progression was defined by the Response Evaluation Criteria in Solid Tumors, RECIST v.1.1 [22]. Progression-free survival (PFS) was defined as the time from the start of therapy to progression or death from any cause, whichever occurred first. Patients with no tumor progression or death at time of data collection were censored at the last date of evaluation. Time to strategy failure (TTSF) was defined as the interval from the start of first-line therapy to progression on full therapy or death. PFS and OS were estimated using Kaplan–Meier method with Rothman’s 95% confidence intervals (CI) and compared across the groups using the log-rank test. Neutrophilia was defined as ≥7500 neutrophils/mm^3^; thrombocytosis was defined as ≥400,000 platelets/mm^3^, while corrected hypercalcemia as ≥10.2 mg/dL. 

In order to investigate patients’ characteristics predictors of survival, Cox proportional-hazards models were used at univariate and multivariate analyses. All the significance levels were set at a 0.05 value and all *p* values were two-sided. The statistical analysis was performed by MedCalc version 11.4.4.0 (MedCalc Software, Broekstraat 52,9030 Mariakerke, Belgium). 

## 5. Conclusions

Our results support the prognostic role of Hb levels in patients treated with cabozantinib and the importance of sequencing immunotherapy and targeted therapy. Further perspective studies should be provided in order to validate these prognostic factors and compare the sequential approaches available in this disease.

## Figures and Tables

**Table 1:** Patients’ characteristics. Immunotherapy combinations included axitinib plus pembrolizumab, axitinib plus avelumab and nivolumab plus ipilimumab.IMDC—International Metastatic Renal Cell Carcinoma Database Consortium.

Clinicopathological Features	N. of Patients (%)
**Age**	
Median	62.56y
Range	24.55–85.76y
**Gender**	
Male	174 (73.42)
Female	63 (26.58)
**T-Stage at Diagnosis**	
T1	37 (15.61)
T2	35 (14.77)
T3	97 (40.93)
T4	26 (10.97)
Unknown	42 (17.72)
**Histology**	
Clear-cell RCC	182 (76.79)
Non-clear-cell RCC	55 (23.21)
**Fuhrman or WHO/ISUP Grade**	
Grade 1	4 (1.69)
Grade 2	62 (26.16)
Grade 3	86 (36.39)
Grade 4	32 (13.50)
Unknown	59 (22.36)
**N. of Metastatic Sites at Recurrence**	
1 site	77 (32.49)
≥2 sites	160 (67.51)
**Site of Metastasis**	
Lung	154 (64.98)
Lymph nodes	133 (56.12)
Bone	80 (34.04)
Liver	53 (22.36)
Brain	20 (8.44)
**IMDC Risk Group**	
Good	57 (24.05)
Intermediate	146 (61.60)
Poor	34 (14.35)
**First-Line Therapy**	
Sunitinib	141 (59.49)
Pazopanib	81 (34.18)
Immunotherapy combinations	9 (3.80)
Other	6 (2.53)
**Second-Line Therapy**	237 (100)
Cabozantinib	112 (47.26)
Nivolumab	89 (37.55)
Axitinib	19 (8.01)
Everolimus	14 (5.91)
Other	3 (1.27)
**Third-Line Therapy**	178 (100)
Cabozantinib	125 (70.22)
Nivolumab	29 (16.29)
Other	24 (13.49)

**Table 2:** Distribution of risk factors according to IMDC criteria in the study populations. LLN = lower limit of normal; ULN = upper limit of normal.

IMDC Criteria	N of Patients (%)
**<1 y from Diagnosis to Systemic Therapy**	
Yes	120 (50.63)
No	117 (49.37)
**Performance Status < 80% (Karnofsky)**	
Yes	19 (8.02)
No	214 (91.98)
**Hb Level < LLN**	
Yes	88 (37.13)
No	149 (62.87)
**Calcium Level > ULN**	
Yes	21 (8.86)
No	216 (91.14)
**Neutrophil > ULN**	
Yes	29 (12.24)
No	208 (87.76)
**Platelets > ULN**	
Yes	31 (13.08)
No	206 (86.92)

**Table 3:** Progression-free survival and overall survival obtained by cabozantinib in our study.

Groups	Second-Line Cabozantinib		Third-Line Cabozantinib	
**All Patients**	PFS [Median (95% CI)]	OS [Median (95% CI)]	PFS [Median (95% CI)]	OS [Median (95% CI)]
	7.76 (6.51–10.88)	11.57 (10.90–NR)	11.38 (5.79–NR)	NR (11.5–NR)
	Second-line Cabozantinib (29 patients, 25.9%)		Third-line Cabozantinib (28 patients, 22.4%)	
**Favourable Group**	PFS [Median (95% CI)]	OS [Median (95% CI)]	PFS [Median (95% CI)]	OS [Median (95% CI)]
	11.28 (7.89–NR)	12.53 (11.57–NR)	11.38 (4.24–NR)	NR (7.40–NR)
	Second-line Cabozantinib (64 patients, 57.1%)		Third-line Cabozantinib (78 patients, 68.4%)	
**Intermediate** **Group**	PFS [Median (95% CI)]	OS [Median (95% CI)]	PFS [Median (95% CI)]	OS [Median (95% CI)]
	7.59 (5.52–NR)	10.95 (9.11–NR)	7.63 (5.56–NR)	NR (11.51–NR)
	Second-line Cabozantinib (19 patients, 17.0%)		Third-line Cabozantinib (19 patients, 9.2%)	
**Poor-Risk Group**	PFS [Median (95% CI)]	OS [Median (95% CI)]	PFS [Median (95% CI)]	OS [Median (95% CI)]
	7.13 (2.66–NR)	11.05 (7.46–NR)	5.75 (3.19–NR)	NR (4.01–NR)
